# The Organisation of Ebola Virus Reveals a Capacity for Extensive, Modular Polyploidy

**DOI:** 10.1371/journal.pone.0029608

**Published:** 2012-01-11

**Authors:** Daniel R. Beniac, Pasquale L. Melito, Shauna L. deVarennes, Shannon L. Hiebert, Melissa J. Rabb, Lindsey L. Lamboo, Steven M. Jones, Timothy F. Booth

**Affiliations:** 1 Viral Diseases Division, National Microbiology Laboratory, Public Health Agency of Canada, Winnipeg, Manitoba, Canada; 2 Department of Medical Microbiology, University of Manitoba, Winnipeg, Manitoba, Canada; 3 Department of Microbiology, University of Manitoba, Winnipeg, Manitoba, Canada; College of Medicine, Hallym University, Korea

## Abstract

**Background:**

Filoviruses, including Ebola virus, are unusual in being filamentous animal viruses. Structural data on the arrangement, stoichiometry and organisation of the component molecules of filoviruses has until now been lacking, partially due to the need to work under level 4 biological containment. The present study provides unique insights into the structure of this deadly pathogen.

**Methodology and Principal Findings:**

We have investigated the structure of Ebola virus using a combination of cryo-electron microscopy, cryo-electron tomography, sub-tomogram averaging, and single particle image processing. Here we report the three-dimensional structure and architecture of Ebola virus and establish that multiple copies of the RNA genome can be packaged to produce polyploid virus particles, through an extreme degree of length polymorphism. We show that the helical Ebola virus inner nucleocapsid containing RNA and nucleoprotein is stabilized by an outer layer of VP24-VP35 bridges. Elucidation of the structure of the membrane-associated glycoprotein in its native state indicates that the putative receptor-binding site is occluded within the molecule, while a major neutralizing epitope is exposed on its surface proximal to the viral envelope. The matrix protein VP40 forms a regular lattice within the envelope, although its contacts with the nucleocapsid are irregular.

**Conclusions:**

The results of this study demonstrate a modular organization in Ebola virus that accommodates a well-ordered, symmetrical nucleocapsid within a flexible, tubular membrane envelope.

## Introduction

Viruses have evolved as genome packaging machines to efficiently transfer nucleic acids between susceptible host cells, ensuring replication. The majority of viruses have hollow, quasi-spherical shells rather than tubular structures, perhaps because this gives the most efficient packaging of nucleic acid with a fixed copy number of coat protein subunits. In non-enveloped viruses, the volume enclosed by the (usually) icosahedral structure is a constraint on the size of the genome, giving a limited capacity to encode capsid proteins, and usually restricts the genome copy number, or ploidy of the virion, to one [1]. Most membrane-enveloped viruses are also quasi-spherical, but their symmetry is frequently less well-ordered, which is usually described as pleomorphic. This feature allows some flexibility in volume, which could accommodate variation in the size of the genome or its copy number. Nevertheless, most viruses, irrespective of their architecture, appear to have evolved to encapsidate only a single copy of their genome within the protein or protein/lipid shell, or a dimeric copy in retroviruses. Notable exceptions are the *Paramyxoviridae* and the *Birnaviridae* where particles may contain up to four copies of the RNA genomes [2], [3]. Although some strains of influenza can produce elongated virions, there is a mechanism that selectively encapsidates only one set of genome segments in each virion [4].

The *Filoviridae* family, including the *Ebolavirus* and *Marburgvirus* genera, cause haemorrhagic fevers with high mortality in humans, and no effective treatments are currently approved [5], although candidate vaccines are promising [6]. The 18.9 kb single-stranded negative-sense non-segmented RNA genome of Ebola virus (EBOV) codes for at least eight proteins. The ribonucleoprotein complex is composed of the nucleoprotein (NP), polymerase protein (L), VP24, VP30, and VP35. The trimeric transmembrane glycoprotein (GP) forms surface spikes on the virion envelope and also has a soluble form, while the matrix protein, VP40, is associated with the inner surface. [5], [7]–[9]. The GP spike, a class I fusion protein, mediates cellular attachment and entry and is extensively glycosylated, especially in the glycan-rich mucin-like domain [10]–[13]. Three proteins, VP24, VP35 and NP are essential for nucleocapsid formation [14]. Although some of the major protein interactions that occur during EBOV morphogenesis have been characterised [14], [15] , the three-dimensional (3D) structure and molecular arrangements have not been previously determined. Structural details are essential to understand how protection of the genome, cell binding, entry, and immune evasion are achieved in a filamentous animal virus, and to determine how this unique morphology plays a role in pathogenesis.

Research on filoviruses has been hampered by their status as biosafety level 4 pathogens. Previous investigations of filovirus structures within embedded, sectioned and metal-stained cells by electron tomography revealed few details of the high resolution oligomeric structure [16], [17]. It has been demonstrated that aldehyde-fixation alone, and subsequent cryo-electron microscopic imaging in the frozen-hydrated state preserves structures, at least up to 12 angstroms resolution [18], and in some cases, fixation improves the resolution achievable [19]. In addition, it has also been shown that high-resolution X-ray structures can also be obtained in the presence of aldehyde fixatives [20]. Therefore, we analyzed purified and isolated EBOV and Ebola virus-like structures using cryo-electron microscopy (cryo-EM), and cryo-electron tomography (cryo-ET). In the current study, the Zaire strain of EBOV was purified and inactivated by paraformaldehyde fixation: excess fixative was then removed by dialysis to reduce beam damage for imaging in the frozen-hydrated state. The flash-freezing at liquid ethane temperatures used in cryo-electron microscopy preserves the structural and molecular detail, avoiding artifacts associated with conventional EM methods, such as dehydration and/or sectioning or staining, that usually prevent detailed structural analysis. Digital image processing reveals the 3D organization of EBOV, including the structural arrangement of component molecules at resolutions of 14–19 Å.

## Results and Discussion

### Structure of EBOV

**Figure 1:**
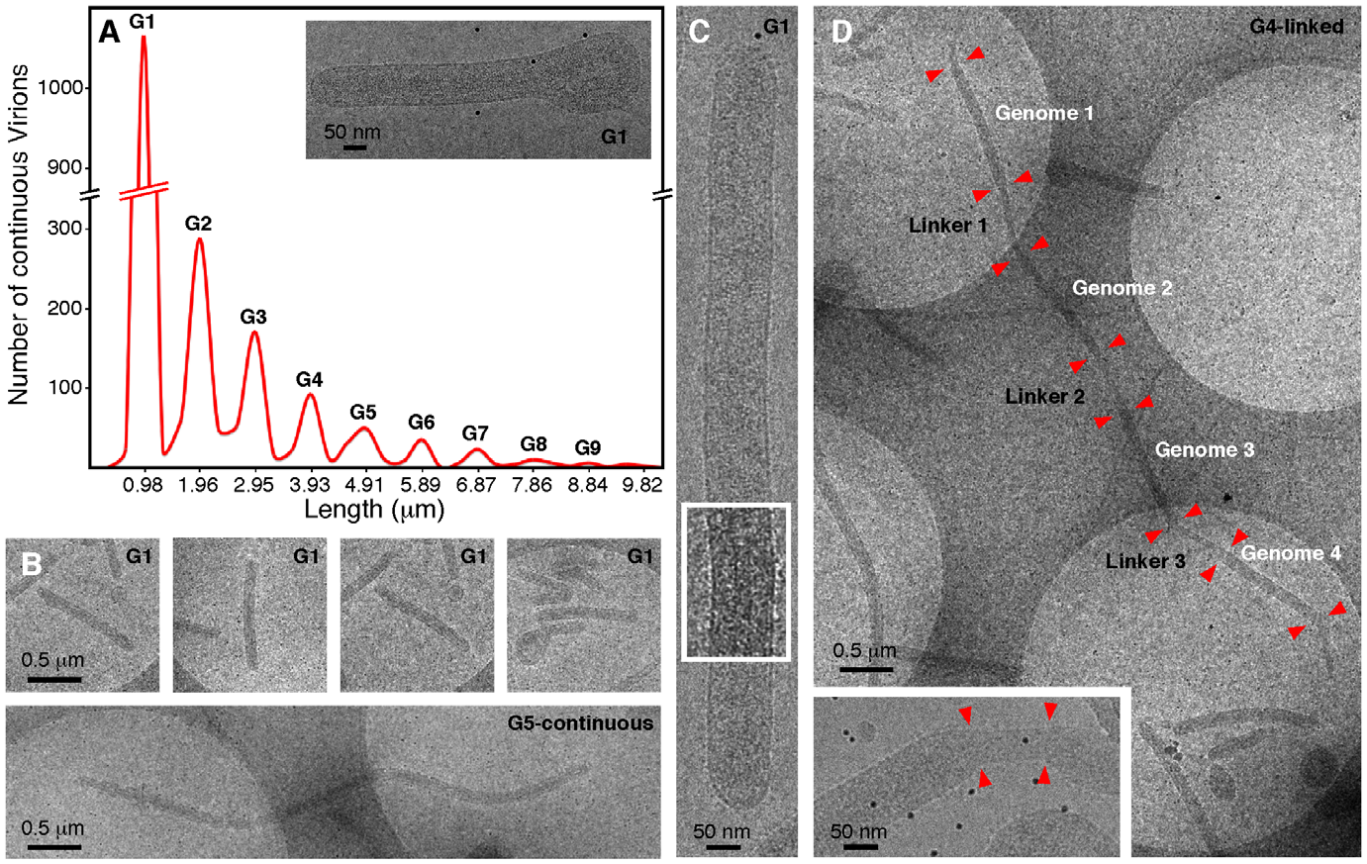
Quantitation of Ebola virus length. (A) Histogram of virion length, with cryo-EM images showing single, continuous and linked particles. A total of 2090 virions with continuous nucleocapsids (no obvious segmentations) were measured, showing the relationship between length and genome copy number per virus. Empty and linked EBOV structures were excluded from the histogram data. A single G1-single/comma shaped EBOV is shown (inset on the right, G1 = 1 copy of genome). (B) Low magnification cryo-images showing: G1- single/comma shape, G1- single/linear, G5-continuous (G5 = 5 copies of genome). (C) High magnification of a G1- (single genome) virion with a region filtered to emphasize the nucleocapsid. (D) Low magnification image of a G4-linked EBOV, each genome copy is indicated and numbered, the red arrows show the transition points between nucleocapsids. The circular holes (filled with vitreous ice) appear as lighter regions and the support film (“quantifoil”) appears dark grey. A “linker” region is shown at higher magnification (inset).

**Figure 2:**
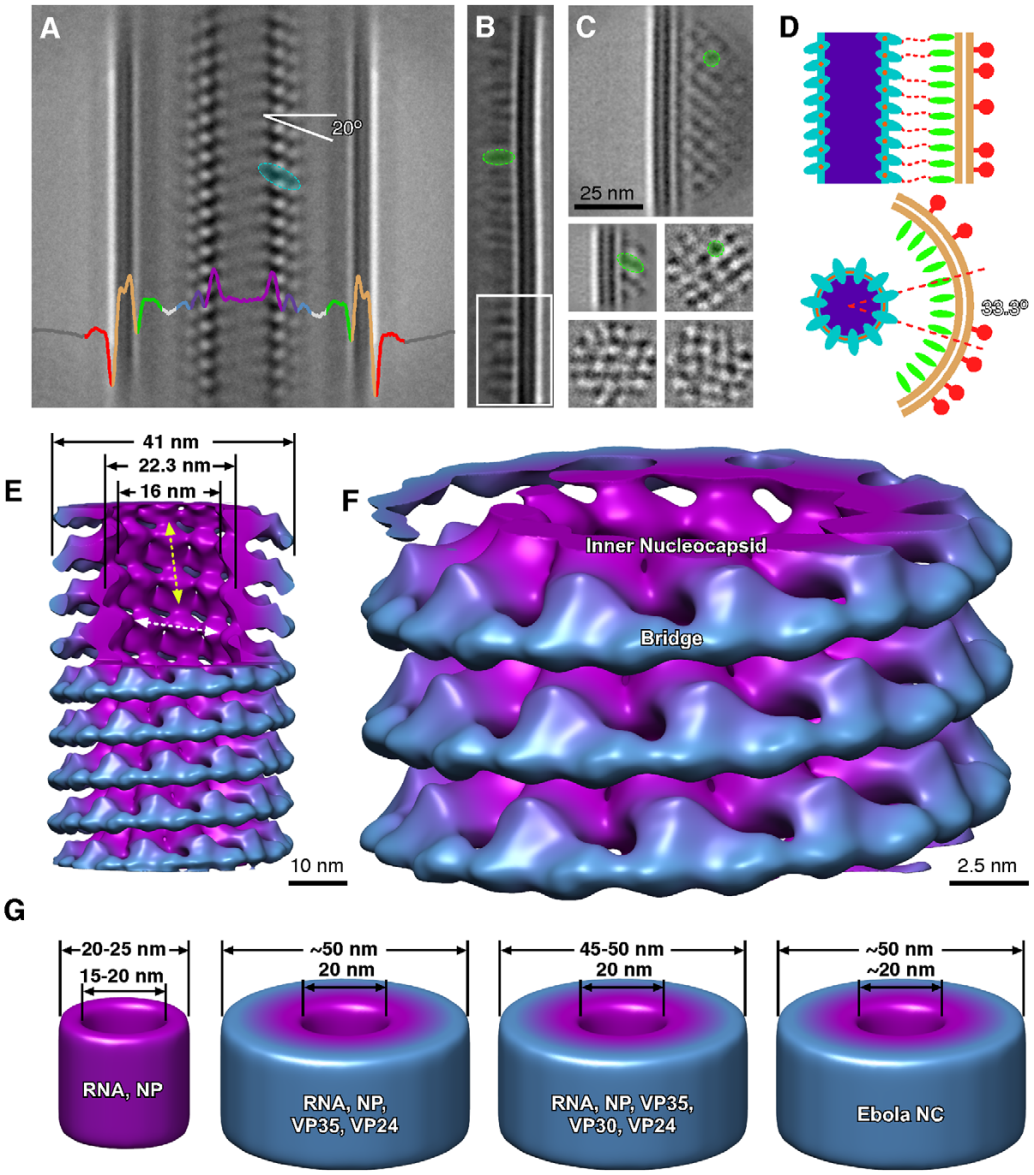
Image processing of Ebola virus. Linear 2D averaging of EBOV: the envelope and nucleocapsid are prominent features (A). The line trace is colour-coded as follows: red, spike; beige, lipid envelope; green, membrane-associated proteins; white, membrane-nucleocapsid gap; blue and purple, outer and inner nucleocapsid. (B) 2D class averages of envelope plus inner face. (C) VP40 VLPs, showing 2D averages from the from side regions (first two) and end-on/central regions (last three). In (A–C) representative individual repeats have been highlighted in color using the same scheme as in (A). (D) Schematic model of the nucleocapsid and envelope, highlighting the relative distribution of NP to VP40. (E,F) 3D reconstruction of the nucleocapsid with the same colour scheme as in (A). The location of the inner nucleocapsid, and the bridge are indicated. The reconstruction is presented at a volume threshold that would encompass a single copy of each of these proteins, and the viral RNA. In (E) the vertical (protein-protein) and horizontal (protein-RNA) contacts are indicated by yellow and white arrows, respectively. (G) Various recombinant nucleocapsid-like structures, and authentic EBOV, which have been studied by electron microscopy [14], [15], [21], [28]. 3D schematics of these structures highlighting the RNA and protein composition and the diameter of these structures, at the same scale for comparison to (E).

**Figure 3:**
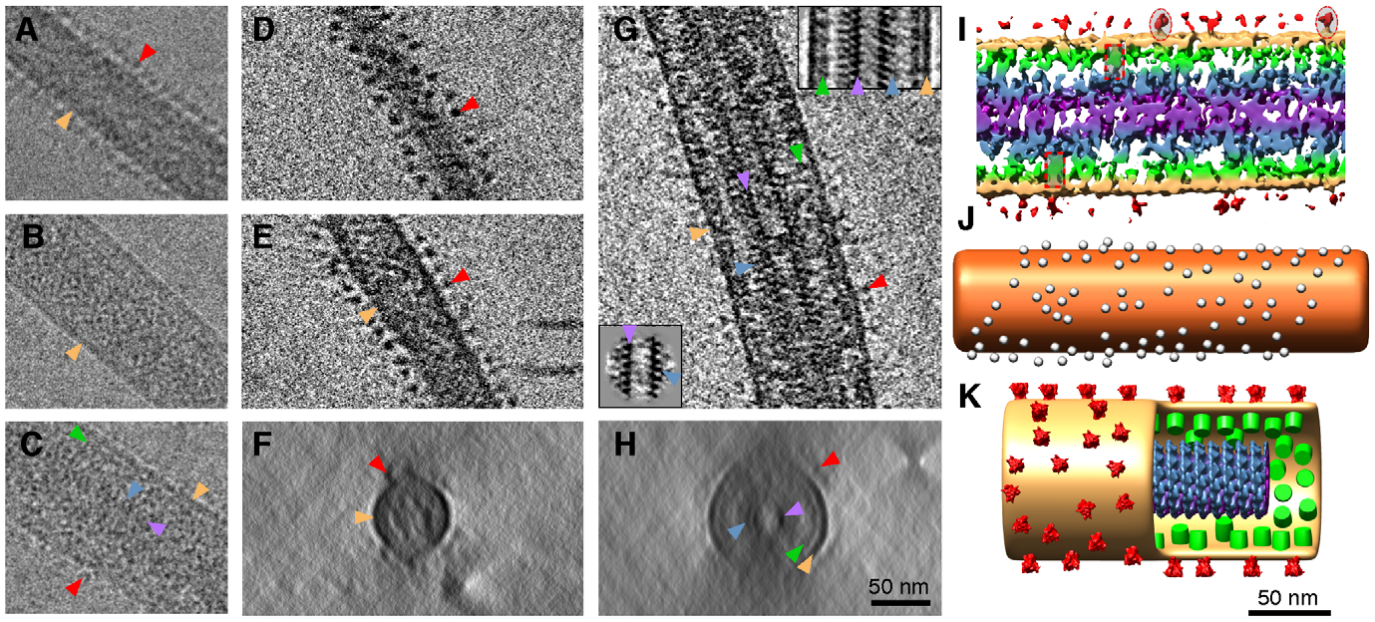
Electron tomography of Ebola virus. (A–C) Cryo-EM images of; (A) VP40-GP VLP, (B) VP40 VLP, and (C) EBOV. (D–F) Electron tomogram of VP40-GP VLP showing; (D) a single X–Y slice cutting through the spikes at the top of the VLP, (E) a single X–Y slice cutting through the central region of the VLP, and (F) a X–Z average of 50 slices showing a cross section of the VLP. (G–H) Electron tomograms of EBOV showing; (G) a single X–Y slice cutting through the central region of the virus, and (H) a X–Z average of 50 slices showing a cross section of the virus. The insets in (G) are image averages of the nucleocapsid (bottom), and the entire width of the virus (top). (I) 3D shaded surface representation of the EBOV tomogram, individual spikes (red oval) and connective regions between the nucleocapsid and membrane proteins (red rectangle) have been highlighted. (J) Surface distribution of the spikes on the VP40-GP VLP tomogram shown in (D–F), the envelope has been replaced with an orange cylinder, and spike locations are indicated by white spheres. (K) 3D model of EBOV using the data from Figs. 2, 3, 4. Color coding as follows: red, spike; white spheres, spike location; beige/orange, lipid envelope; green, membrane associated proteins; blue and purple, outer and inner nucleocapsid. Colour-coded arrows in (A–H) highlight equivalent features in the 2D analysis shown in Fig. 2.

**Figure 4:**
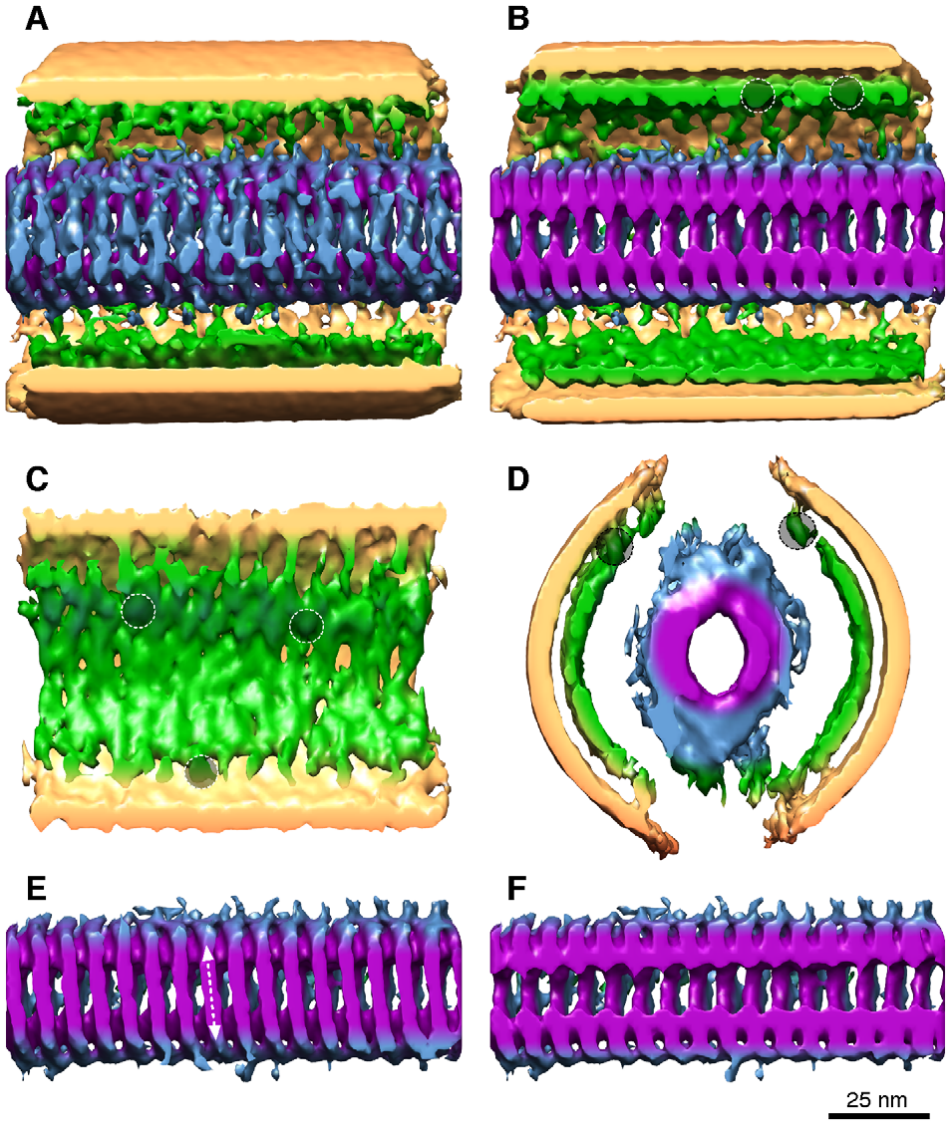
Sub-tomogram averaging of Ebola virus. (A–D) Sections of the density map of the sub-tomogram average are shown from the top sliced just below the envelope (A), the middle of the virus (B), a side view of the virus (C), and an end-on slice (D). Putative locations of several VP40 proteins adjacent to the membrane are circled. (E,F) Images showing just the nucleocapsid. The helix is right handed (arrow in E). No helical symmetry was applied to this data. Color coding as follows; beige, lipid envelope; green, membrane associated proteins (VP40); blue and purple, outer and inner nucleocapsid.

**Figure 5:**
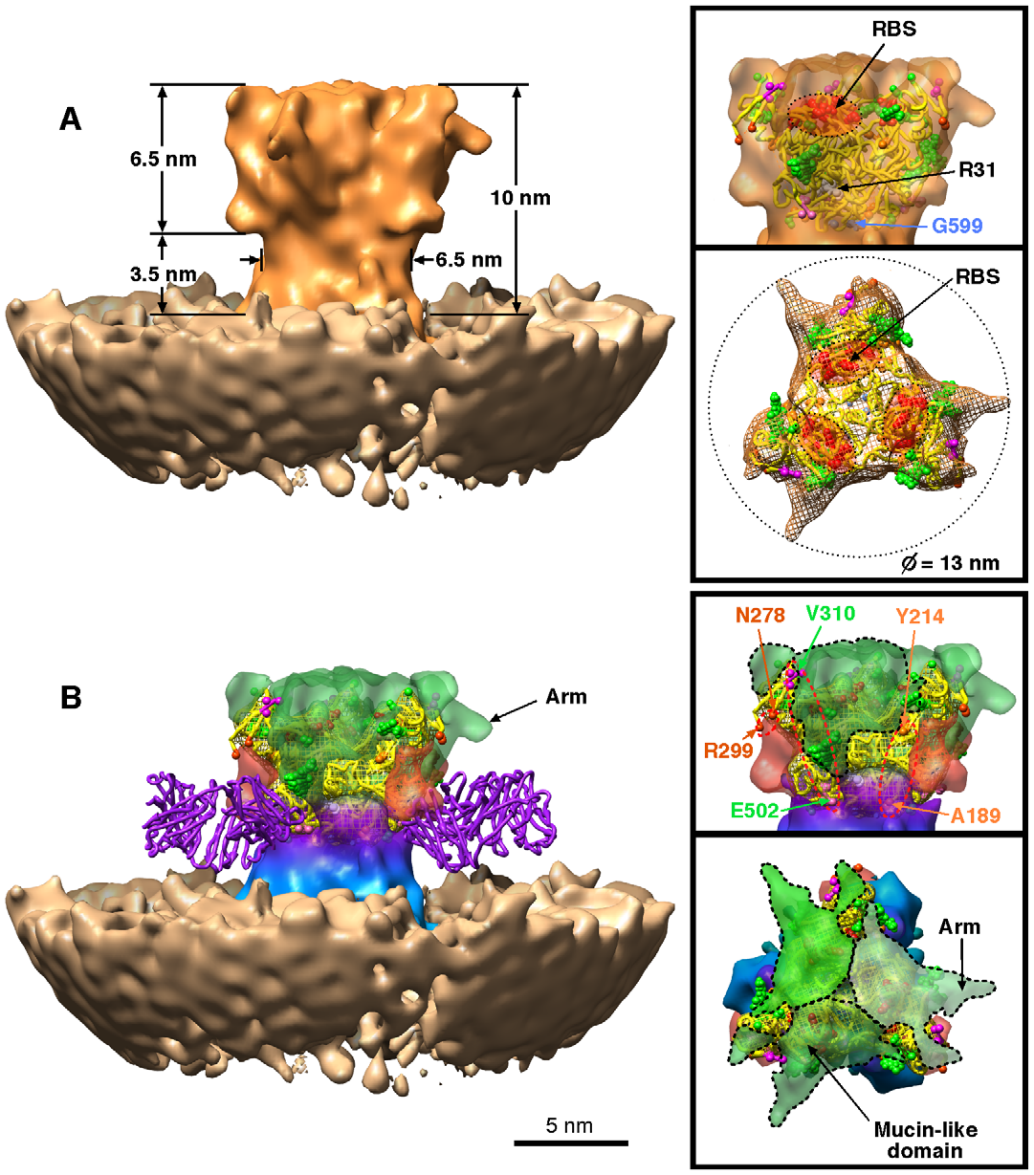
3D structure of the Ebola spike. The density map of the EBOV GP spike viewed from the side, end-on, and side (with envelope) shows the docked GP1–GP2 structure in yellow (PDB entry 3CSY [42]), glycosylation sites (green), and receptor binding site (RBS; red, highlighted). (A) The reconstruction showing the spike (orange) and the envelope (beige). (B) Difference map generated by subtracting the docked structure from reconstruction of the entire spike. The color scheme shows the following putative regions; green, mucin domain; pink, deletions 190–213, 279–298; purple-blue, GP2 stalk. The docked KZ52 neutralizing antibody is shown in purple.

**Figure 6:**
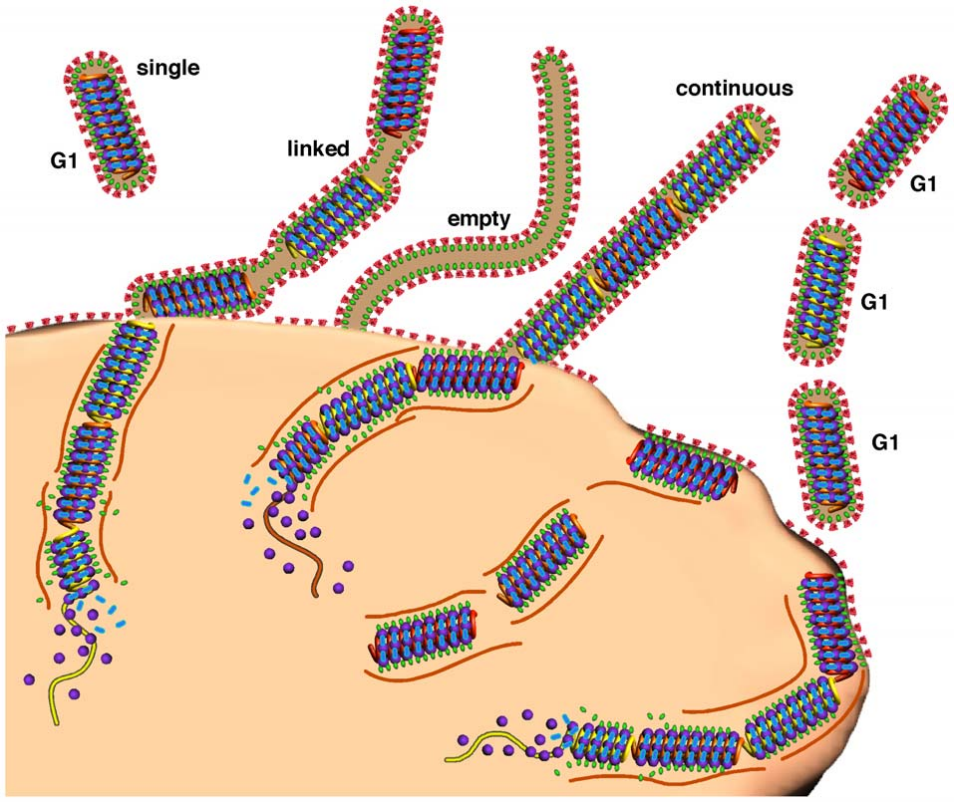
Schematic model of Ebola virus genome packaging. EBOV appears pleomorphic, but an underlying structural organization is maintained. In the model we show the three basic morphological forms of EBOV particles; empty, linked, and continuous. Single genome (G1) virus and multi-genome particles are shown budding from the cell. In this model genomes are assembled in the host cell and transported to the surface where the end-to-end apposition that we have observed by cryo-EM in mature virions takes place during (or prior to) budding and envelopment at the plasma membrane. The color-coding is as follows: nucleocapsids, red, yellow and orange helices; nucleocapsid protein, purple spheres; VP40, green ovals, VP24/VP35 bridges, blue oval; GP spikes, red; microtubules, brown.

We identified filamentous EBOV particles 20 microns or longer, with a well ordered internal structure, and a helical nucleocapsid giving an internal “herring-bone” appearance using cryo-EM and cryo-ET (Figures 1, 2, S1, S5). The nucleocapsid, as observed within intact viral particles, has a uniform helical structure (Figures 1, 2, 3, 4) and is enveloped by a membrane coated by an external layer of GP spikes. From the same image data set, we combined extracted volumes from tomograms with 2-D single particle processing to determine the structure of the GP spikes (Figure 5) to a resolution of 14 Å as measured by the Fourier Shell Correlation (FSC) 0.5 criterion. Virions are rarely straight. Variation in the overall length of virions is non-random, and they fit into ordered size classes (Figure 1). Analysis of 2090 distinct intact virions with a nucleocapsid from cryo-electron micrographs shows that the most common class length (53%) of virus particles is 982±79 nm (Figure 1A, Table S1). The other size classes are multiples of this length. Enveloped filovirus particles have several different morphologies (shown in Figures 6, S1, S5). These configurations are “single” particles, containing a nucleocapsid of uniform length, (which we postulate to contain one copy of the genome), “continuous” particles, with nucleocapsids of a length of the single virion multiplied by an integer of 2 or greater; and “linked” virions composed of a series single-genome nucleocapsids connected by short sections of empty envelope. Negative staining can cause drying and staining artefacts which hamper accurate measurements and preclude 3D analysis. Nevertheless, in previous studies using this technique, the average length of Marburg virus was measured as 790 nm, and EBOV as 970 nm [21], [22]: the latter is very similar to the mean length of 982 nm measured in the current study by cryo-TEM of frozen hydrated specimens. In addition, discrete sub-populations of virions of double or triple the average length were also previously reported in centrifuged virus preparations [21].

We also observed “empty” filaments that lack nucleocapsids, with a random length and a smaller diameter (Figure S1C). These empty nucleocapsids are also visible in previously published thin-section micrographs of human EBOV infected pathology specimens, e.g. [23], and are thus probably not an artifact of cell culture. It is also unlikely that the polyploidy we have observed in EBOV is a peculiarity of the particular viral isolate or of the Vero cell line that we used for this investigation. Previous studies using negative stain EM have shown that both Marburg virus and several different EBOV species can produce filamentous virions up to 14 µm long when grown in several different cell lines [21], [22]. Blood specimens from Guinea-pigs and monkeys that were inoculated with primary isolates of the Marburg agent, when centrifuged and observed by negative stain, also showed filamentous virions of variable length, with the average reported as about 1 µm in length but with a smaller proportion being over 2 µm in length [24]. In addition, viral filaments of lengths from 1.4 to 1.6 µm long, and occasionally as long as 2.6 µm, can also be seen in published ultrathin sections of EBOV infected human and monkey tissues [23], [25]. Thin-section EM always underestimates the lengths of virions, since filament profiles are frequently truncated by the section plane: in addition, tissue shrinkage of 10 to 20% during dehydration and resin embedding is common. Thus, it is probable that polyploid EBOV with multiple nucleocapsids are also produced in naturally infected humans and animals, though it is not possible to make a direct comparison with cell cultured virus: it would be very difficult to obtain large enough quantities of concentrated virus from animals to carry out detailed cryo-TEM analysis and measurements.

### Nucleocapsid

The Ebola nucleocapsid structure was solved to 19 Å resolution (FSC 0.5 criteria) using linear regions of 34,605 images (taken at 3–4 µm defocus, Figure 2). Since virus particles were not straight enough for conventional helical image processing, a combination of tomography, sub-tomogram averaging, single particle averaging, and the iterative helical real-space construction method were used [26], [27]. The EBOV nucleocapsid is a right-handed double-layered helix with an outer diameter of 41 nm and a hollow inner channel 16 nm in diameter as determined by image analysis and tomography (Figures 2, 3, 4, S2, S4, movie S1). The pitch is 6.96 nm, with 10.81 repeats per helical turn (Figure 2). The inner nucleocapsid, composed of large subunits, is linked by vertical and horizontal contacts between the large subunits (Figure 2E,F). The horizontal contacts occur between the large subunits at a diameter of 22.3 nm. The vertical contacts linking the coils have a higher density than the horizontal ones, so we interpret the horizontal contacts as involving viral RNA (white arrow, Figures 2E, 4E; movie S1) and the vertical contacts as protein-protein interactions (yellow arrow, Figure 2E). The NP subunits are also linked by an outer horizontal layer at a diameter of 37 nm. This layer consists of a ring of bridges between adjacent large subunits (Figure 2). The bridges joining the NP subunits are composed of two lobes, one of which of which is slightly bigger than the other. Previous studies that produced recombinant nucleocapsid-like structures showed that expressed VP24 and VP35 both independently associate with NP, but that all three proteins together are necessary to produce ∼50 nm diameter helical nucleocapsid-like structures. When VP35, VP30, VP24, and NP were transfected together, approximately 50 nm diameter helical nucleocapsid-like structure was also generated, whereas NP alone generated helical NP-RNA complexes ∼20–25 nm in diameter, which were nuclease sensitive [14], [28], [29]. Taken together these results suggest that VP24 and VP35 are the structural components of the bridge located on the periphery of the nucleocapsid (Figure 2G). It is not possible to accurately delineate VP24 and VP35 within the bridge at this resolution, however the density of the bridge is consistent with a predicted total mass of 60 kD, thus each bridge is composed of one molecule of VP24 and one molecule of VP35. It is likely that the larger lobe is VP35 and that VP24 therefore resides within the smaller lobe (Figure 2F). Thus, each bridge is composed of a VP24-VP35 heterodimer that holds adjacent NP molecules together horizontally. This structure explains how VP24 and VP35 are able to independently interact with NP, and why all three are required for the formation of a double-layered nucleocapsid. In addition, it implies that VP24 and VP35 can interact with each other as well as each interacting with a different site on the NP molecule in order to make an oligomeric structure.

The recombinant nucleocapsid data indicates that both VP35-VP30-VP24-NP and VP35-VP24-NP produce approximately 50 nm diameter helical structures that are indistinguishable from nucleocapsids produced by EBOV. This indicates that VP30 does not increase the diameter of the nucleocapsid. We propose that VP30 lies in the interior of the nucleocapsid and is not part of the bridge on the periphery of the nucleocapsid. This localization is consistent with previous work showing that VP30 is a component of the nucleocapsid, and associates with NP, but is non-essential for nucleocapsid formation [14], [30], [31]. Our model, in which the inner layer at 22.3 nm diameter is RNA-NP, and the outer bridge centered at 37 nm is composed of VP24-VP35 heterodimers, with VP30 bound to NP, is thus consistent with these previous observations [14], [28], [29], and suggests that the outer VP24-VP35 heterodimer bridge functions in the stabilization and/or protection of the nucleocapsid.

We have modeled the arrangement of the RNA within the EBOV nucleocapsid (Table S2). Since no atomic resolution data on the ribonucleoprotein structure of filoviruses have yet been reported, the ribonucleoprotein ring-structures of other members of the mononegovirales are useful for comparison. Although the mass of the nucleoproteins, and the number of nucleotides per subunit differs amongst these virus families, a model of the 18.9 kb EBOV genome with the RNA following a circular fixed radius as in respiratory syncytial virus (RSV) [32] would make a nucleocapsid containing one copy of the EBOV genome about 914 nm long (Table S2). This fits closely with the 982 nm length class (53% of the particles observed in this study) containing a single copy of the EBOV genome threaded through the NP at a fixed radius. Allowing 33 nm of space for the membrane envelope to curve around at each end of the virus particle gives an estimated length for a single-genome virus particle of 980 nm, which is very close to that observed (982±79 nm). The majority of the virus particles fall into size categories that are a multiple of the putative single genome length (G1), giving size classes of 1.9±0.15 µm (G2: 18.7% of the particles having 2 genomes); 2.9±0.2 µm (G3: 12% of particles having 3 genomes) and so on (Figure 1A and Table S1) . The length of the longest particle measured was consistent with having 22 genome copies. Our model predicts a nucleotide to NP ratio of 13.0 (Table S2), which is within the range of 12 to 15 nucleotides per NP molecule as measured by biochemical studies of Marburg virus [33].

Viral filaments containing a nucleocapsid have a diameter of 96 to 98 nm, while empty viral filaments are 48 to 52 nm in diameter. In partially full virions, the membrane envelope is constricted at the transition point where the nucleocapsid ends and the empty membrane tube begins (Figures 1, S1C). Empty virions (Figure S1C) have a similar structure to VP40-GP virus-like particles (Figure 3A). Both full and empty virions have a continuous layer of spikes projecting from the surface (Figures S1, S2, S3, movie S2), giving an overall diameter of 120 nm for full particles. The majority of virions are linear (Figures 1, S1A, S5), others have a “comma-shaped” appearance, with a globular head containing portions of the nucleocapsid that are curled-up or bent at one end (Figures 1A,B and S1B). It is clear that “toroidal” virions previously identified by negative staining [22], [34] are a variation of comma-shaped virions. Internal vesicles of 20–40 nm in size are also sometimes observed at the ends of virions (Figure S1D). These vesicles appear to be formed during the process of envelopment of the nucleocapsid, since they were not observed in preparations of VP40 or VP40-GP VLPs. VP40 VLPs had wavy envelopes with an irregular diameter ranging between 48 nm and 142 nm (N = 49) compared to VP40-GP VLPs which were more ordered with a diameter between 50 nm and 91 nm (N = 26), (Figure 3). Thus the presence of GP, and certain contacts between the GP and VP40, play a part in stabilizing the tubular membrane envelope structure, and our observed structure agrees with previous reports that GP enhances VP40 VLP budding [35], [36]. The previously reported “branched”, filamentous forms [34] were rarely observed: these consist of empty tubes (data not shown). It is possible that centrifugal virus purification disrupted most branched structures that were seen previously with negative staining of cell culture supernatant.

The VP40 matrix protein shows a regular 5 nm lattice spacing (Figure 2B, C). Both the nucleocapsid and the VP40 layers are ordered, however the contacts between them appear to be non-symmetrical (Figure 2D), implying some flexibility in their inter-molecular contacts. It has been shown that VP35 interacts with the VP40 and can be packaged into VP40 VLPs [37]. The localization of the VP24-VP35 bridges on the periphery of the nucleocapsid, may allow interactions of one or more of the nucleocapsid proteins with VP40, possibly through projecting low-density protein loops. There is a 6–7 nm gap of low density between the nucleocapsid and the VP40 layer, but tomography also shows discrete areas of connectivity between the nucleocapsid and matrix protein layers, which may be connections between the envelope and the nucleocapsid (Figure 3I). These results are substantiated by the analysis of sub-tomograms where helical symmetry is clearly evident but was not imposed (Figures 4, S4). The use of sub-tomogram analysis improves the resolution of the data by averaging sub-tomograms together which are at different angular orientations. The helical nucleocapsid is clearly identified in the structure, including the gap between the envelope-VP40, and the nucleocapsid-VP40. The right-handed pitch of the helix is clearly discernable (Figure 4E), as well as the putative location of several VP40 proteins, although the resolution of the tomographic data by itself is slightly lower than with single particle analysis.

While showing the overall organization of EBOV, tomography also allows estimation of the stoichiometry of the major structural proteins (Figure 3, movie S2). Both EBOV and VP40-GP VLPs have an irregular distribution of the GP spikes on the surface (Figures 3, S3) demonstrating the lack of any ordered lattice-like arrangement with the matrix protein VP40. The spikes are clustered, and the average centre-to-centre spacing is 15.2 nm (Figure S3, and movie S3). We calculate that a virion of 982 nm in length would have about 1888 copies of the GP spike, and 8391 copies of VP40. The wide spacing of the EBOV GP in the viral envelope allows plenty of space for free access and binding of any neutralizing antibodies directed at both the club-shaped head and stem region without stearic hindrance (Figures 3J, S3).

The nucleocapsid structure implies equimolar ratios of NP, VP24, VP30, and VP35. A genome of 18.9 kbp with 13 bases per NP with a single genome copy gives 1454 molecules of NP, VP24, VP30, and VP35 per virion. Previous analyses of Coomassie blue stained gels of purified EBOV predicted 625, 1208, 833, and 2686 protein molecules of NP, VP24, VP30, and VP35, per virion respectively [38], which is in the same stoichiometric range as predicted by our model for the nucleocapsid, taking into account the variability of individual protein band staining by Coomassie blue, which is affected by factors such as distance of migration [39], basic amino acid content [40], and the extent of glycosylation [41]. Since NP is glycosylated, we anticipate underestimation of the NP content of virions [14], [41].

### GP spike

The GP spike is necessary for cellular attachment and fusion of EBOV. A definitive cell surface ligand for the receptor binding domain has not been identified, and a number of different cell surface proteins are able to enhance infection [42], [43]. Cleavage of GP results in two domains: GP1 containing the receptor binding domain and GP2 that contains the fusion and transmembrane domains. The structure of a smaller engineered fragment of GP1–GP2 has recently been determined by x-ray crystallography (3CSY pdb [42]). We determined the structure of the entire EBOV GP trimeric spike at a resolution of 14 Å (FSC 0.5 criteria), by combining sub-tomograms from the spikes of VP40-GP VLPs (234 images) with EBOV images for the side perspective data (8084 images) using projection matching as previously described [44] (Figure 5 and movie S4). In our reconstruction, the spike is *in situ* in the viral membrane, thus the transmembrane region and base of the spike, adjacent to the membrane, are less well defined than the distal region of the spike, due to the smaller differences in contrast between lipid and protein versus water and protein, as well as Fresnel fringes at the edge of the viral membrane. The spike extends 10 nm from the surface of the envelope, with a club-shaped head 6.5 nm in height, and a 3.5 nm long stalk. Docking of the previously determined X-ray structure into our cryo-EM map shows a good fit, with a correlation coefficient of 0.75 using the docking and correlation program SITUS [45]. The difference map calculated between our cryo-EM map and the GP1–GP2 structure identified volumes corresponding to the domains deleted to generate the 3CSY GP1–GP2 structure (Figure 5). We show that the mucin-like domains (connected at V310 and E502 - shown in Green in Figure 5) completely fill the previously described bowl-like chalice which contains the putative receptor binding sites [42]. The proximity of glycosylation sites in the GP1–GP2 structure suggests that the distal density of the spike contains the glycans that were deleted in order to construct the GP1–GP2 structure. In addition, each mucin-like domain has an “arm-like” projection, which extends radially at the distal end of the spike, to a maximum diameter of 13 nm. The localization of the mucin-like domain is consistent with previous studies showing that endosomal proteolysis plays a role in enhancing infectivity as well as binding of the Ebola GP to the plasma membrane [46], [47].

The other two major deletions in the 3CSY structure (N278-R299 and A189-Y214) are situated at the midpoint of the structure just above the stalk (shown in pink in Figure 5). Inclusion of the KZ52 Fab in the docked structure demonstrates that this neutralizing epitope (from a human survivor) is localized on the side of the stem region of EBOV GP trimer at the base of the club-shaped head, and that the Fab domain lies close to the lipid envelope when bound, and approximately tangential to the viral envelope. The densities putatively corresponding to N278-R299 and A189-Y214 are close to the KZ52 neutralizing site, but do not obstruct antibody binding. The mucin-like domains are out of the way and cannot interfere stearically with KZ52 Fab binding. This is consistent with previous results indicating that KZ52 binding does not require cathepsin cleavage [48]. We have thus delineated the low resolution structure of the glycocalyx or “glycan cap” that covers the distal end of the uncleaved EBOV GP spike, which is consistent with a proposed role in immune evasion [42]. Our data will enable docking of future structures to investigate receptor binding, antigenicity, and fusion mechanisms.

We have shown that EBOV particles are capable of a high degree of polyploidy, made possible by the extreme length polymorphism of budding virus particles. Polyploidy in filoviruses may be more extensive than in any other virus family, with 46% of virions having more than one genome copy, and some having up to 22 copies. Polyploidy has been shown to increase infectivity rates in paramyxovirus and birnavirus [2], [3]. Attempts to investigate infectivity rates of EBOV by centrifugal fractionation of the different sized particles are stymied by the extreme filamentous morphology, as well as that fact that EBOV of different lengths have the same buoyant density (personal observations). A complex double layered helical nucleocapsid appears to be unique to filoviruses. In the case of rhabdoviruses, the bullet-shaped nucleocapsid precludes them from being linked sequentially. Although some influenza strains can produce filamentous virions, this morphology appears to be driven by the matrix protein only [49]. A filamentous morphology may have evolutionary implications by allowing genome length flexibility. It could also enhance the ability for viral dissemination in infected tissues, for example by diapedesis of budding filamentous virions through epithelial layers.

## Materials and Methods

### Cells and viruses

Zaire Ebolavirus was propagated in Vero E6 cells and purified as previously described [50]. Ebola enriched samples were checked by SDS-Page and Western blotting, and rendered non-infectious by fixation with 4% paraformaldehyde. Excess fixative was removed by placing the fixed samples in a Slide-A-Lyzer G2 cassette with a 0.5 ml capacity, and a 10,000 MWCO (Thermo Scientific Pierce Protein Research Products, Rockford, Illinois, USA), followed by dialysis against PBS. Virus-like particles were produced as previously described [51]. All work with infectious Ebola virus (virus culture and purification) was performed in the biosafety level 4 laboratories at the National Microbiology Laboratory of the Public Health Agency of Canada, Winnipeg, Manitoba.

### Cryo-electron microscopy

Samples for cryo-electron microscopy (cryo-EM), and cryo-electron tomography (cryo-ET) were mixed with BSA coated 10 nm gold particles (Aurion Immuno Gold Reagents & Accessories, Wageningen, The Netherlands) at a ratio of 2∶1 (virus∶gold) for cryo-ET, and (9∶1) for cryo-EM. Specimens (4 µl) were then applied to glow-discharged quantifoil grids with 2 µm holes spaced at 1 µm intervals (Quantifoil MicroTools GmbH, Jena, Germany). Grids were subsequently plunge cooled in liquid ethane using a Vitrobot Mark IV (FEI Company, Hillsboro, Oregon, USA). Specimens were transferred to a Tecnai 20 G2 transmission electron microscope (FEI) operated at 200 kV, equipped with a Gatan CT3500TR single tilt rotation low-temperature specimen holder. For cryo-EM imaging was conducted at temperatures of ∼−185°C. Images were recorded using an Eagle 4K CCD camera (FEI Company, Hillsboro, Oregon, USA). For single particle image analysis, images were taken at 50,000× or 80,000× magnification at 2–4 µm defocus, with a dose of 10 electrons/Å^2^. This corresponded to a pixel size at the CCD detector of 2.147 Å/pixel and 1.353 Å/pixel, respectively. For virus length measurements low magnification cryo-EM images were taken at 5,000×, 3,500× and 2,500×. For cryo-ET single axis tomograms were taken at 25,000×, 29,000× or 50,000× magnification, at −8 µ or −6 µ defocus, with angle steps of 2°−4°. Data were collected within tilt ranges of ±60°, or ±52°, with a total dose/tomographic data set of 47–60 electrons/Å^2^. For cryo-EM, data collection was done using the low-dose unit and software coupled with the TEM Imaging & Analysis (TIA) software (FEI Company, Hillsboro, Oregon, USA). Automated eucentricity determination, and focusing were performed using the Xplore3D data acquisition software (FEI Company, Hillsboro, Oregon, USA). For cryo-ET, data collection was done using the Xplore3D data acquisition software, the low-dose unit, and the TIA software (FEI Company, Hillsboro, Oregon, USA).

### Image processing: virus length measurements

The exact magnification in the microscope at the CCD detector was determined using a calibration grid (Pelco International, Redding, CA). Ebola virus length measurements (n = 2090) were made using the Image J software package [52] using the free hand line tool, and the analyse/measure function. For this analysis only viruses containing a continuous nucleocapsid were measured. Viruses with linked nucleocapsids and empty viruses were omitted. The measurements that were made in image J were then collated, analysed, and plotted, using Microsoft Excel.

### Tomography: image processing

Tomographic image analysis of cryo-ET data was carried out with the Inspect3D Xpress software package (FEI Company, Hillsboro, Oregon, USA). The tomographic images were aligned to each other by a two-step process. The first step involved alignment of adjacent images by cross correlation. This process was repeated several times until the shift between adjacent images was below one pixel in either the X or Y plane. The second step involved the alignment of the entire image stack using 10 nm colloidal gold particles as fiducial markers. In this instance the term “image stack” refers to all the images collected in a single tomographic tilt. This procedure involves the selection of ten to twenty of the 10 nm gold particles and the subsequent identification and tracking of these particles in all of the images in the image stack. The locations of these particles are then used in conjecture with the tilt angles of each image to globally align all of the images to each other. The last step in this process was to calculate the three dimensional reconstruction of the tomogram from the aligned images. In this study we used the simultaneous iterative reconstruction technique (SIRT) algorithm with 10 iterations to calculate the final three-dimensional reconstruction (tomogram).

### Sub-tomogram analysis

Sub-tomogram image analysis of cryo-ET data was carried out with the Automated Recognition of Geometries, Objects, and Segmentations (ARGOS) software package (FEI Company, Hillsboro, Oregon, USA). For this analysis an 80^3^ pixel sub-tomogram was extracted from a tomogram using the Chimera [53] software package. In this analysis the tomogram used contained a linear region of the Ebola virus (Figure S4), and the 80^3^ pixel sub-tomogram contained a single linear segment. This template was then used by the ARGOS software to conduct an exhaustive search of the original tomogram for similar structures. This analysis involved a six dimensional search matrix (three positional variants, and three rotational variants). The entire search process was sped up by the ARGOS software by utilizing parallel processing on the computer's graphics processing unit (GPU). Once individual sub-tomograms were selected based on correlation, they were inspected and compared to the initial template sub-tomogram. The extracted and aligned sub-tomograms were subsequently averaged with a filter that minimized the missing wedge artifact. This average structure then was used as the reference and the entire procedure was repeated several times.

### Single particle image analysis: software and hardware

Single particle cryo-EM image processing was carried out using the EMAN/EMAN2 and SPIDER/WEB image processing program packages [54], [55]. Particle selection (EMAN) and contrast transfer function correction (EMAN2) were conducted on an Apple Inc. Mac Pro computer (12-core, Intel Xeon Nehalem processors 2.93 GHz, 32 GB Ram, Mac OS X 10.6.7). All subsequent calculations were performed on a Dell PowerEdge R900 4-way 64-bit Xeon X7460 processors, Six Core 2.67 GHz CPUs with 256 GB Ram running Linux (CentOS 5.2). Images were corrected for contrast transfer function (ctf) using the “e2ctf.py” function in the EMAN2 software package^5^, which estimates defocus and corrects for ctf by phase-flipping. Images of the spike (n = 8084 side perspective; n = 234 end-on perspective) and nucleocapsid (n = 34,605) were selected for image analysis. The resolution of the cryo-EM reconstruction was estimated by Fourier shell correlation using the FSC 0.5 criteria. In all subsequent sections image analysis procedures were conducted using the SPIDER software package unless otherwise stated.

### Single particle image analysis: nucleocapsid analysis

Analysis of initial images of the “straight” linear segments of the Ebola virus using Fourier transformation indicated that there was sufficient bending of the helical nucleocapsid to make standard helical analysis problematic. Therefore, an initial reference free single particle 2D analysis was conducted in EMAN using the “startnrclasses” program to identify any potentially recurring motifs within linear regions of the Ebola virus.

In order to further investigate the nucleocapsid repeat identified in the 2D analysis the iterative helical real space reconstruction method (IHRSR) was implemented [26], [27]. This procedure requires an initial 3D helical reference structure which is used for image alignment. In this investigation a linear region of the Ebola virus which was extracted from a tomogram was used to generate this helical reference structure. This initial 3D structure was first pre-treated with a Gaussian mask to select only the nucleocapsid-containing region of the virus tomogram. An auto correlation function was then performed in which the volume was rotated around the helical axis, and translated along the axis of the nucleocapsid. At each rotational and translational position and autocorrelation value was calculated (between the shifted and un-shifted volume). The net result of this process was the determination of the helical symmetry present in the tomogram. In order to analyse the data generated by this procedure the Microsoft excel spread sheet program was used. The correlation plots generated by this process solved the handedness, pitch, and number of repeats per turn for the nucleocapsid. These symmetry parameters were then applied to the tomogram to generate the initial 3D model. The IHRSR method was then applied to the 34,605 single particle images of the nucleocapsid as previously described [26], [27].

### Single particle image analysis: spike analysis

For the spike dataset two image populations were combined composed of side, and end-on perspectives. For the side view perspective images were subfield directly off of the Ebola virus cryo-EM images. For the end-on perspectives sub-tomographic volumes were extracted from the tomographic reconstructions of the Ebola VLP. The 3D volumes were then added in the “Z” plane to generate 2D projection averages which were then used for the subsequent single particle image analysis. The data were the processed using EMAN to generate an initial 3D reconstruction, which was then refined in SPIDER using the projection matching technique as previously described [44], [56]. The docking of the 3CSY.pdb [42] structure to the cryo-EM structure of the spike was accomplished using the SITUS [57] software package with the exception that only the GP1 and GP2 components of the 3CSY structure were used for the docking process. The “floodfill” program in SITUS was used to segment the spike component of the 3D cryo-EM reconstruction from the envelope component of the reconstruction. The segmented volume was then used for the docking procedure using the ‘colores’ function in SITUS. Once docked the entire 3CSY.pdb structure which included the Fab of the KZ52 neutralizing antibody was superposed over the docked GP1/GP2 component of the structure.

### Structure visualisation

The 3D cryo-EM reconstructions, cryo-ET reconstructions, 3D models of the Ebola virus, and the atomic resolution structure 3CSY.pdb were visualized using UCSF Chimera software package (Computer Graphics Laboratory, University of California, San Francisco, supported by NIH P41 RR-01081) [53]. The 3D images and movies presented in this manuscript were generated directly by UCSF Chimera software package.

## Supporting Information

Figure S1 **High magnification images showing linear regions of Ebola virus.** The diameter of EBOV is constant in linear regions of the virus containing a nucleocapsid (A). The viral filaments are not perfectly straight, and are often curved, complicating helical image processing. Glycoprotein spikes (GP), envelope (E), and nucleocapsid (NC) are all clearly visible. The black spherical objects (shown by black arrows) are 10 nm colloidal gold particles which are used for automated focusing and tomography alignment. (B) Images of “comma-shaped” Ebola virus. Each of the comma-shaped viruses has a single copy of the genome which can be seen running through the center of the virus and curving at one end of the virus to form the globular head. In some of the heads there is a low-density region (LD) devoid of nucleocapsid. In others, the nucleocapsid is sharply bent where it folds back on itself and looks like a check mark (right panel). (C) Ebola virus structures with and without nucleocapsid. Empty tubular filaments (E), and viral filaments containing a nucleocapsid (NC) are shown. Constrictions at the transition points where the nucleocapsid ends and the viral membrane continues as an empty tubular structure are indicated by red arrow heads. The diameters are as follows: nucleocapsid, 41 nm; virus with nucleocapsid, 96–98 nm; empty filaments, 48–52 nm. (D) Ebola virus with interior vesicles. Viral particles containing additional membrane vesicles within the envelope are shown by a green “V”. Although present in a minority of virus particles, when present they are usually at the ends of the virus with a globular head. The image in the left hand column shows an example where the vesicles are in the middle of the virus.
(TIF)

Figure S2 **Tomographic slices of Ebola virus.** Slices in “Z” of Ebola virus tomogram reveal the components of the virus and nucleocapsid (A). The insets show 2D averages of the nucleocapsid. The slice through the top of the virus reveals the envelope and glycoprotein spikes. The next slice cuts through the top of the nucleocapsid (NC) revealing the banding pattern which represents VP24–VP35 bridge. The third slice cuts through the middle of the NC. The tube-like component of the NC is primarily composed of NP. The last panel shows the average of 53 slices which make up the volume encompassing the entire NC. The image and the average in the inset contain all structural components of the NC. (B) The linear region of the tomographic nucleocapsid reconstruction (A) was translated in Z and rotated 360° in plane. At each shift/rotation point the volume was correlated to the initial (un-shifted) volume. The correlation plot is shown as a grey scale image. Eight regions have been highlighted demonstrating a characteristic right-handed helical pattern. (C). For comparison, both left and right handed helical correlation plots are shown. (D), Region three is shown, with the locations of correlation maxima shown with an X. The angular distance between each maximum was calculated from several plots. A total of 71 measurements gave an average angular distance of 33.6°+/−8.5° between helical repeats, resulting in 10.7 repeats per turn. Using the 6.96 nm pitch (Fig. S8) the step in Z per helical repeat was calculated as 0.65 nm. These helical symmetry values were then imposed on the nucleocapsid tomogram and this structure was used as the initial reference volume for refinement using the iterative helical real space reconstruction method.
(TIF)

Figure S3 **Surface spike distribution in the Ebola VLP.** Longitudinal Z-slices through the top and middle of the particle are shown, as well as the end-on view (A). The tomogram is shown as a shaded surface at a density threshold that indicates the spikes (B). The volume from one side of the tomogram has been extracted, and a red-blue color scheme shows the depth at which the spikes are located. This region of the envelope has a surface area of 15,651 nm^2^. Selected spikes have been identified by red circles, the single particle reconstruction of the spike is shown at the same scale to the right in a red square for comparison. The same region in (B) is shown in (C) with a solid orange cylinder to provide a visual cue for the viral envelope. Eighty-six individual spikes were counted (white spheres) and have a patchy distribution (D), each spike would occupy an average area of 182 nm^2^, giving an average spacing between spikes of 15.2 nm. The reconstruction of the spike (blue) with the docked KZ52 Fab (purple) has been included to show that there is ample room for antibody attachment.
(TIF)

Figure S4 **Extraction of Ebola nucleocapsid structure for sub-tomographic analysis.** The tomogram of a linear region of the Ebola virus was used as the first reference for sub-tomogram analysis (A). When viewed along the helical axis (Y) or from the end perspectives (X,Z) the basic components are visible. The tomographic volume was also cylindrically masked along the X-axis, selecting only the density containing the nucleocapsid, to highlight the components of the nucleocapsid in the tomogram (B). Two-dimensional single particle image analysis was carried out with cryo-images (C) (not tomographic data sets), for comparison to the 3D tomographic data. The average shown in this panel was generated by reference free classification, using the “startnrclasses” program in EMAN [54]. The 6.96 nm helical pitch can be easily seen in the 2D average, but is also visible in the projections of the tomographic volume in (A, B).
(TIF)

Figure S5 **Representative low-magnification images of Ebola virus.** Frozen hydrated virus is clearly visible with sections of the filamentous virus over both the support film and across the holes in the quantifoil film. Individual G1 (single genome copy) virus is circled in red, several sections containing a nucleocapsid are indicated by a blue arrowhead, and regions without a nucleocapsid are indicated by a magenta arrowhead. Globular heads are identified by yellow arrowheads. In this image the circles (light grey, 2 µ diameter) are filled with frozen hydrated virus in a thin aqueous layer, and the quantifoil support film appears as darker grey.
(TIF)

Table S1 Length analysis of “continuous” Ebola virus particles. The length of 2090 EBOV particles were measured using ImageJ [52]. The values in the “model length” column are based on multiples of the G1 mean length. The values in the in the “mean length” column were calculated directly from the data. Only full particles containing a continuously packaged nucleocapsid were measured, all others (linked-nucleocapsid and empty particles) were omitted from this analysis. The terms G1–G22 indicate the number of genomes/viral particle (i.e. G22 = 22 genomes). All measurements are in µm.
(TIF)

Table S2 Modeling of RNA in the Ebola nucleocapsid. Two previously determined atomic resolution structures of negative stranded RNA viruses (VSV (2GIC.pdb) [58], and RSV (2WJ8.pdb) [32]) were used to estimate the EBOV nucleocapsid length and number of nucleotides per nucleoprotein. Images of VSV (A) and RSV (B) are shown as a molecular surface with the protein in orange and the RNA as a green ribbon. From left to right, they show a surface view from the side, a side-on cross section, an end-on view, the RNA density alone in projection, and a rotational average of the projection. The VSV-based estimate, with a saw-tooth pattern of RNA in the helix, gave a nucleocapsid which 614.37 nm long, too short for the measured length of the G1 EBOV (982 nm). The RSV-based model, with a relatively straight/circular pattern of RNA in the helix predicted a nucleocapsid 914.55 nm long, which closely fits the measured length of G1 virions, after allowing ∼34 nm space at each end to accommodate the curve of the envelope containing GP spikes and matrix proteins. The RSV-like model gives 13 nucleotides per nucleocapsid protein which is similar to previous biochemical estimates of 12–15 for Marburg virus [33], suggesting that the RNA in the EBOV nucleocapsid is arranged in a smooth helical pattern at a diameter of ∼22 nm.
(TIF)

Movie S1 **3-D reconstruction of Ebola virus nucleocapsid.** This movie shows the three-dimensional structure of the of the Ebola virus nucleocapsid as shaded surface representation. The surface is set at a density threshold which would include one copy of NP, VP24,VP30, VP35, and the RNA. The nucleocapsid rotates, and then is sliced through the Z-axis to show the internal components of the structure.
(MOV)

Movie S2 **Tomogram of Ebola virus.** This movie shows slices in “Z” through a cryo-electron tomogram of a linear region of Ebola virus. The slices which are in the Z-plane pass back and forth through the virus showing various components such as the surface glycoprotein spikes and the nucleocapsid in the interior of the virus.
(MOV)

Movie S3 **Ebola virus spike distribution.** This movie shows one surface of a cryo-electron tomogram of an Ebola virus-like particle, generated by expressing the VP40 and GP proteins. The structure rotates showing the distribution of spikes. The locations of individual spikes are identified by white spheres, and the reconstruction is replaced by a cylinder to show the patchy distribution of spikes on the surface of the virus-like particle.
(MOV)

Movie S4 **3-D reconstruction of Ebola virus spike.** This movie shows the three-dimensional structure of the of the Ebola virus GP spike. The structure rotates showing views from different angles, and indicates the location of the docked 3CSY.pdb [42] structure with the GP1 and GP2 domains and KZ52 antibody (purple).
(MOV)
