# Supplementary figures and images for: The Organisation of Ebola Virus Reveals a Capacity for Extensive, Modular Polyploidy
Source: PLoS One. 2012 Jan 11;7(1):e29608. doi: 10.1371/journal.pone.0029608 (PMC3256159; doi:10.1371/journal.pone.0029608)

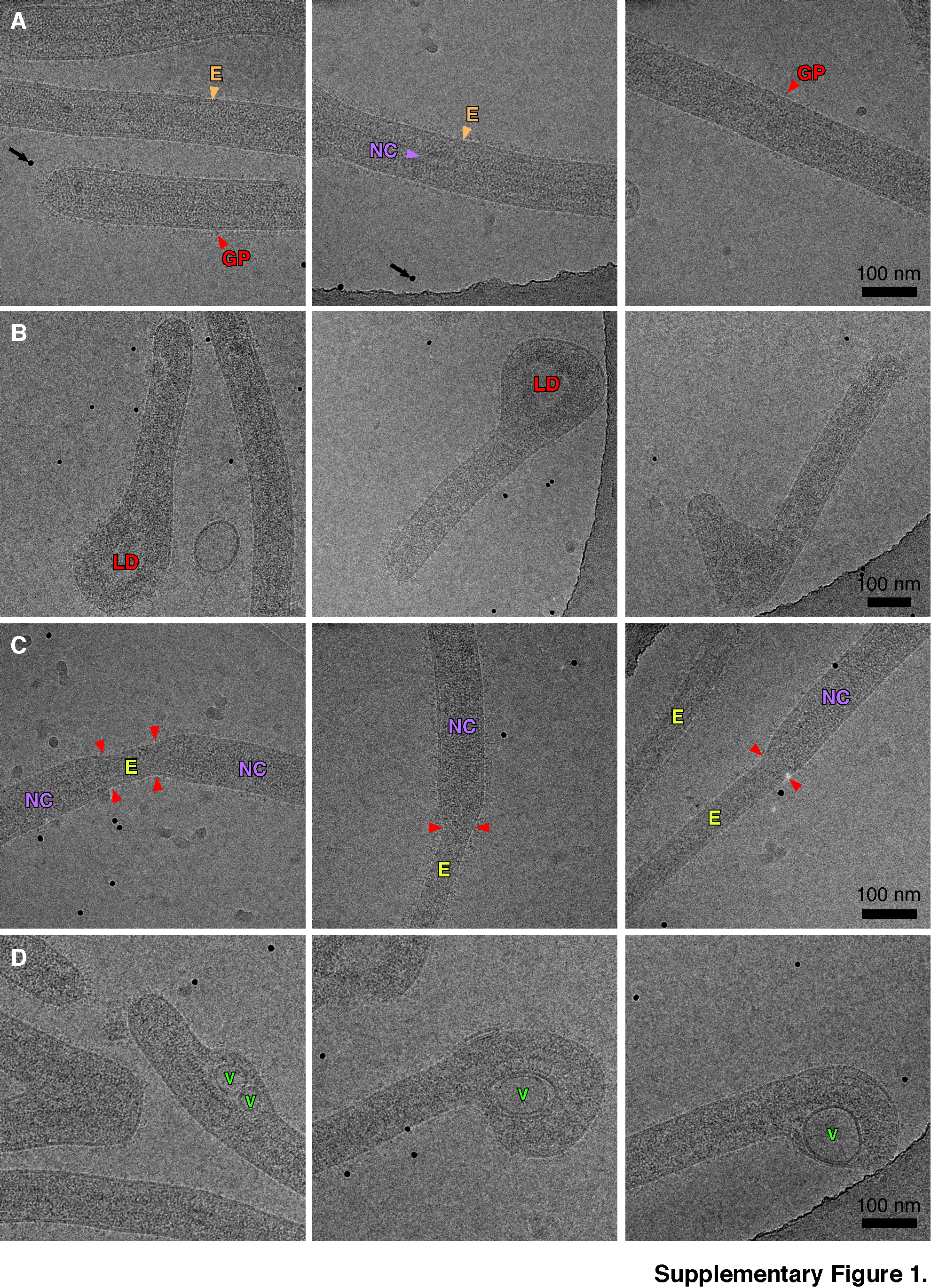

Supplement: Figure S1 — High magnification images showing linear regions of Ebola virus. The diameter of EBOV is constant in linear regions of the virus containing a nucleocapsid (A). The viral filaments are not perfectly straight, and are often curved, complicating helical image processing. Glycoprotein spikes (GP), envelope (E), and nucleocapsid (NC) are all clearly visible. The black spherical objects (shown by black arrows) are 10 nm colloidal gold particles which are used for automated focusing and tomography alignment. (B) Images of “comma-shaped” Ebola virus. Each of the comma-shaped viruses has a single copy of the genome which can be seen running through the center of the virus and curving at one end of the virus to form the globular head. In some of the heads there is a low-density region (LD) devoid of nucleocapsid. In others, the nucleocapsid is sharply bent where it folds back on itself and looks like a check mark (right panel). (C) Ebola virus structures with and without nucleocapsid. Empty tubular filaments (E), and viral filaments containing a nucleocapsid (NC) are shown. Constrictions at the transition points where the nucleocapsid ends and the viral membrane continues as an empty tubular structure are indicated by red arrow heads. The diameters are as follows: nucleocapsid, 41 nm; virus with nucleocapsid, 96–98 nm; empty filaments, 48–52 nm. (D) Ebola virus with interior vesicles. Viral particles containing additional membrane vesicles within the envelope are shown by a green “V”. Although present in a minority of virus particles, when present they are usually at the ends of the virus with a globular head. The image in the left hand column shows an example where the vesicles are in the middle of the virus. (TIF) [file pone.0029608.s001.tif]

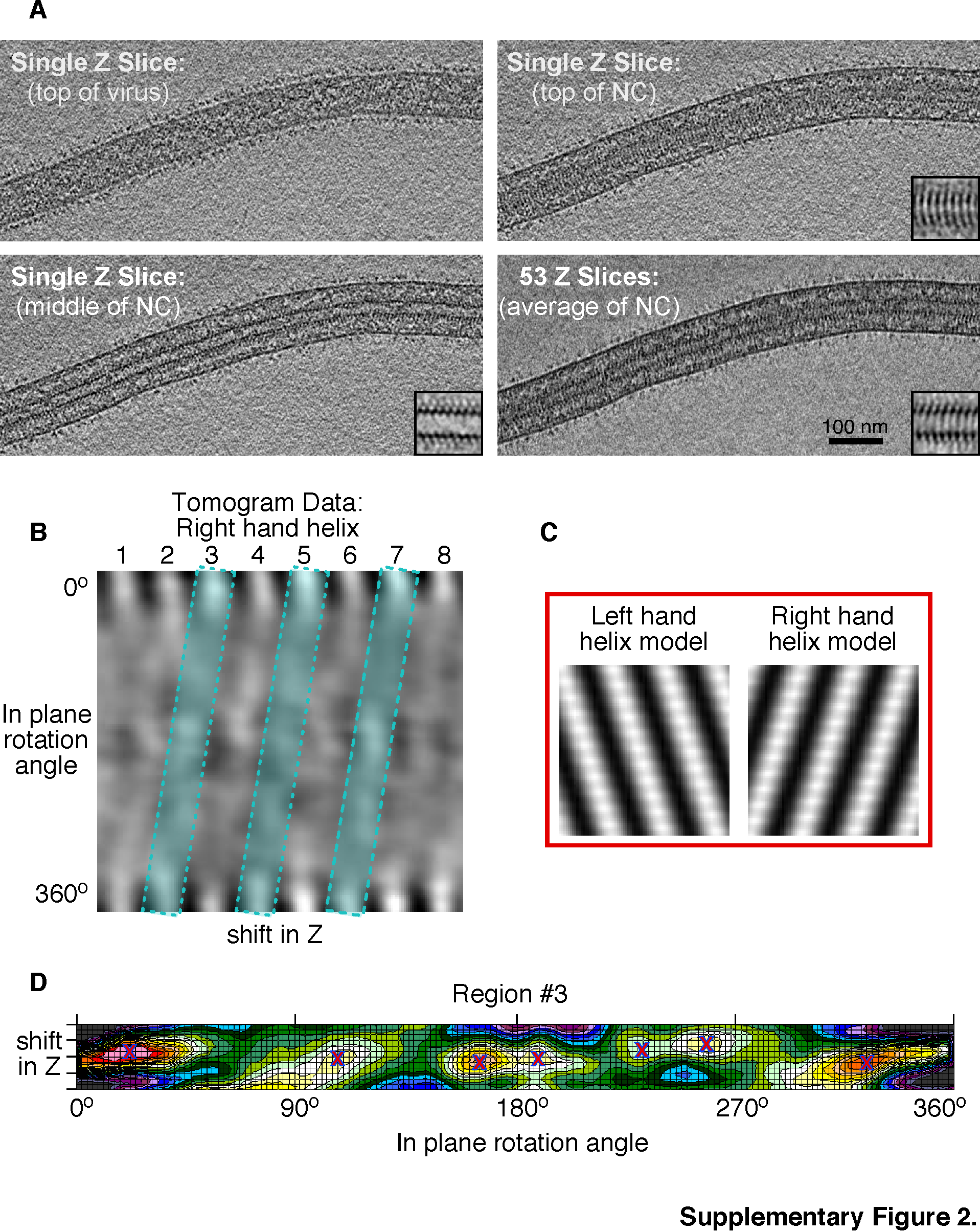

Supplement: Figure S2 — Tomographic slices of Ebola virus. Slices in “Z” of Ebola virus tomogram reveal the components of the virus and nucleocapsid (A). The insets show 2D averages of the nucleocapsid. The slice through the top of the virus reveals the envelope and glycoprotein spikes. The next slice cuts through the top of the nucleocapsid (NC) revealing the banding pattern which represents VP24–VP35 bridge. The third slice cuts through the middle of the NC. The tube-like component of the NC is primarily composed of NP. The last panel shows the average of 53 slices which make up the volume encompassing the entire NC. The image and the average in the inset contain all structural components of the NC. (B) The linear region of the tomographic nucleocapsid reconstruction (A) was translated in Z and rotated 360° in plane. At each shift/rotation point the volume was correlated to the initial (un-shifted) volume. The correlation plot is shown as a grey scale image. Eight regions have been highlighted demonstrating a characteristic right-handed helical pattern. (C). For comparison, both left and right handed helical correlation plots are shown. (D), Region three is shown, with the locations of correlation maxima shown with an X. The angular distance between each maximum was calculated from several plots. A total of 71 measurements gave an average angular distance of 33.6°+/−8.5° between helical repeats, resulting in 10.7 repeats per turn. Using the 6.96 nm pitch (Fig. S8) the step in Z per helical repeat was calculated as 0.65 nm. These helical symmetry values were then imposed on the nucleocapsid tomogram and this structure was used as the initial reference volume for refinement using the iterative helical real space reconstruction method. (TIF) [file pone.0029608.s002.tif]

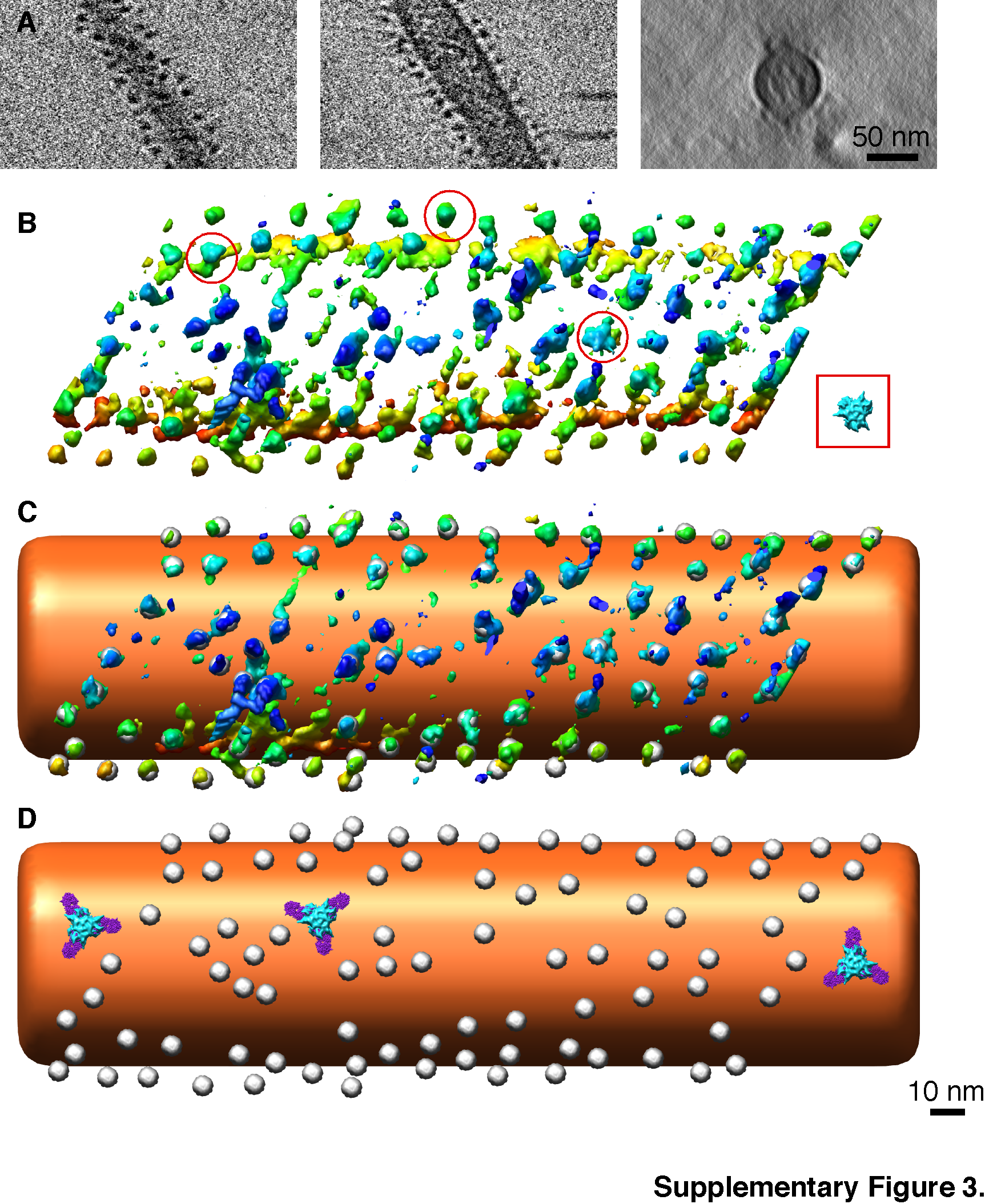

Supplement: Figure S3 — Surface spike distribution in the Ebola VLP. Longitudinal Z-slices through the top and middle of the particle are shown, as well as the end-on view (A). The tomogram is shown as a shaded surface at a density threshold that indicates the spikes (B). The volume from one side of the tomogram has been extracted, and a red-blue color scheme shows the depth at which the spikes are located. This region of the envelope has a surface area of 15,651 nm2. Selected spikes have been identified by red circles, the single particle reconstruction of the spike is shown at the same scale to the right in a red square for comparison. The same region in (B) is shown in (C) with a solid orange cylinder to provide a visual cue for the viral envelope. Eighty-six individual spikes were counted (white spheres) and have a patchy distribution (D), each spike would occupy an average area of 182 nm2, giving an average spacing between spikes of 15.2 nm. The reconstruction of the spike (blue) with the docked KZ52 Fab (purple) has been included to show that there is ample room for antibody attachment. (TIF) [file pone.0029608.s003.tif]

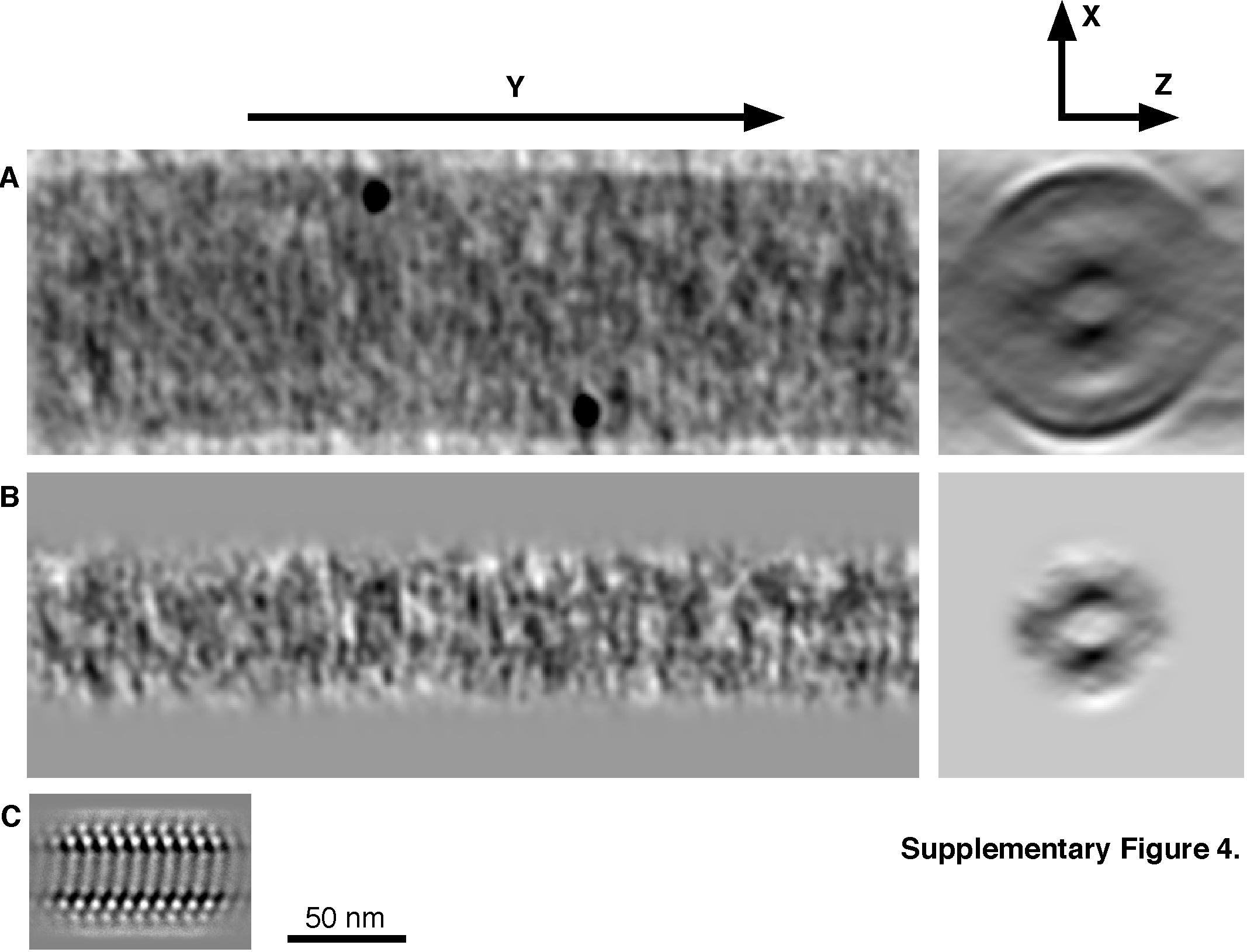

Supplement: Figure S4 — Extraction of Ebola nucleocapsid structure for sub-tomographic analysis. The tomogram of a linear region of the Ebola virus was used as the first reference for sub-tomogram analysis (A). When viewed along the helical axis (Y) or from the end perspectives (X,Z) the basic components are visible. The tomographic volume was also cylindrically masked along the X-axis, selecting only the density containing the nucleocapsid, to highlight the components of the nucleocapsid in the tomogram (B). Two-dimensional single particle image analysis was carried out with cryo-images (C) (not tomographic data sets), for comparison to the 3D tomographic data. The average shown in this panel was generated by reference free classification, using the “startnrclasses” program in EMAN [54]. The 6.96 nm helical pitch can be easily seen in the 2D average, but is also visible in the projections of the tomographic volume in (A, B). (TIF) [file pone.0029608.s004.tif]

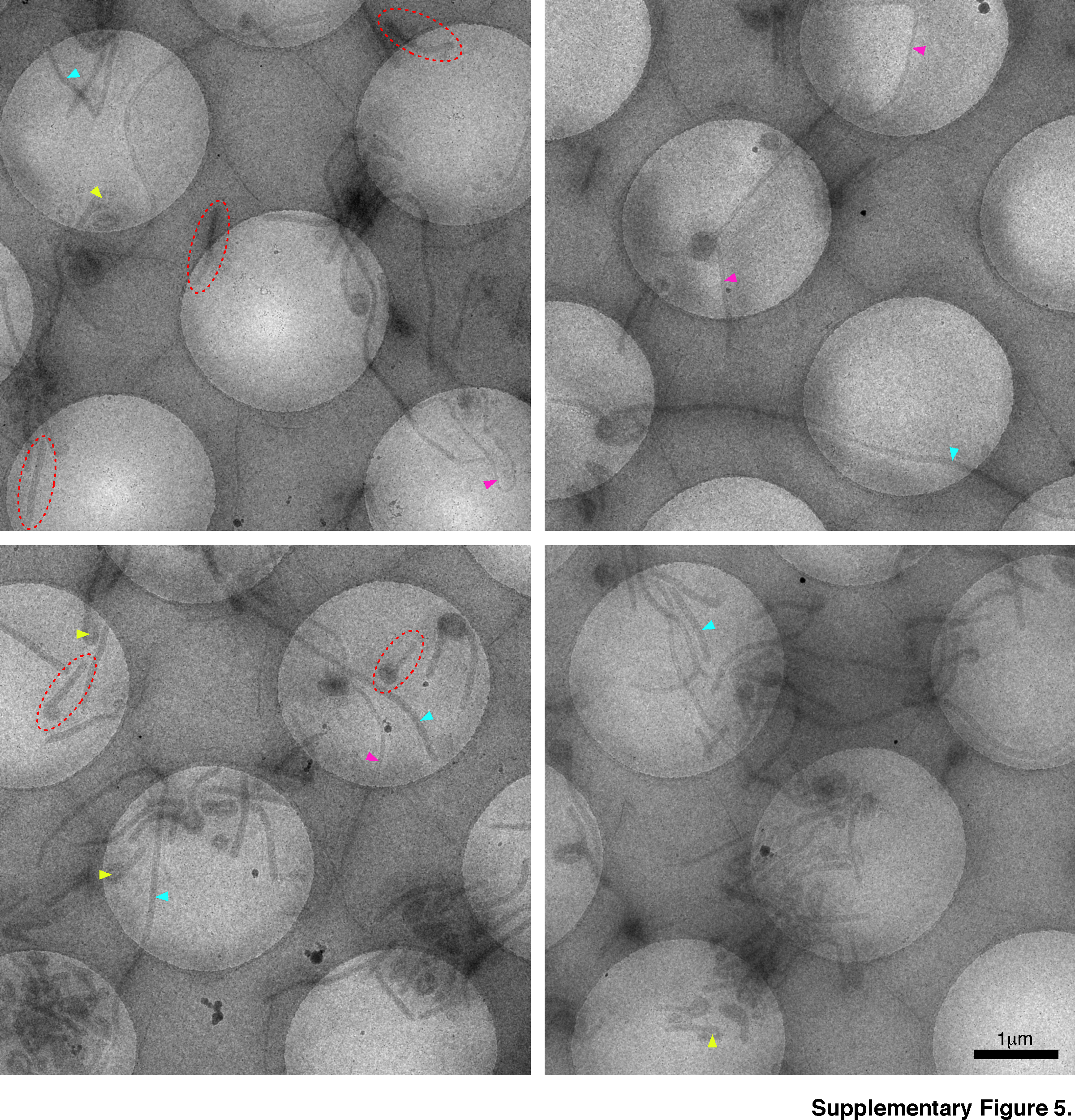

Supplement: Figure S5 — Representative low-magnification images of Ebola virus. Frozen hydrated virus is clearly visible with sections of the filamentous virus over both the support film and across the holes in the quantifoil film. Individual G1 (single genome copy) virus is circled in red, several sections containing a nucleocapsid are indicated by a blue arrowhead, and regions without a nucleocapsid are indicated by a magenta arrowhead. Globular heads are identified by yellow arrowheads. In this image the circles (light grey, 2 µ diameter) are filled with frozen hydrated virus in a thin aqueous layer, and the quantifoil support film appears as darker grey. (TIF) [file pone.0029608.s005.tif]

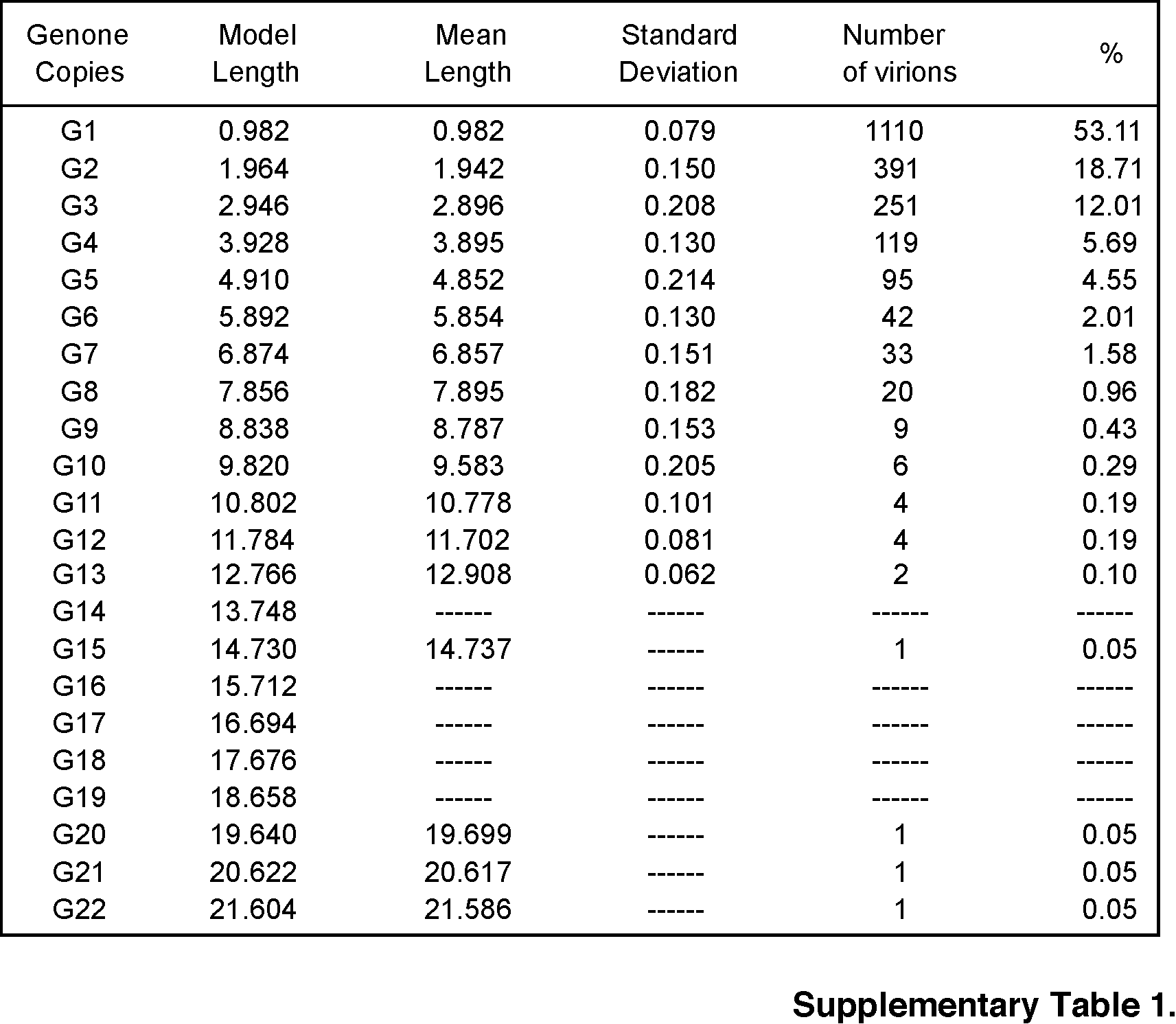

Supplement: Table S1 — Length analysis of “continuous” Ebola virus particles. The length of 2090 EBOV particles were measured using ImageJ [52]. The values in the “model length” column are based on multiples of the G1 mean length. The values in the in the “mean length” column were calculated directly from the data. Only full particles containing a continuously packaged nucleocapsid were measured, all others (linked-nucleocapsid and empty particles) were omitted from this analysis. The terms G1–G22 indicate the number of genomes/viral particle (i.e. G22 = 22 genomes). All measurements are in µm. (TIF) [file pone.0029608.s006.tif]

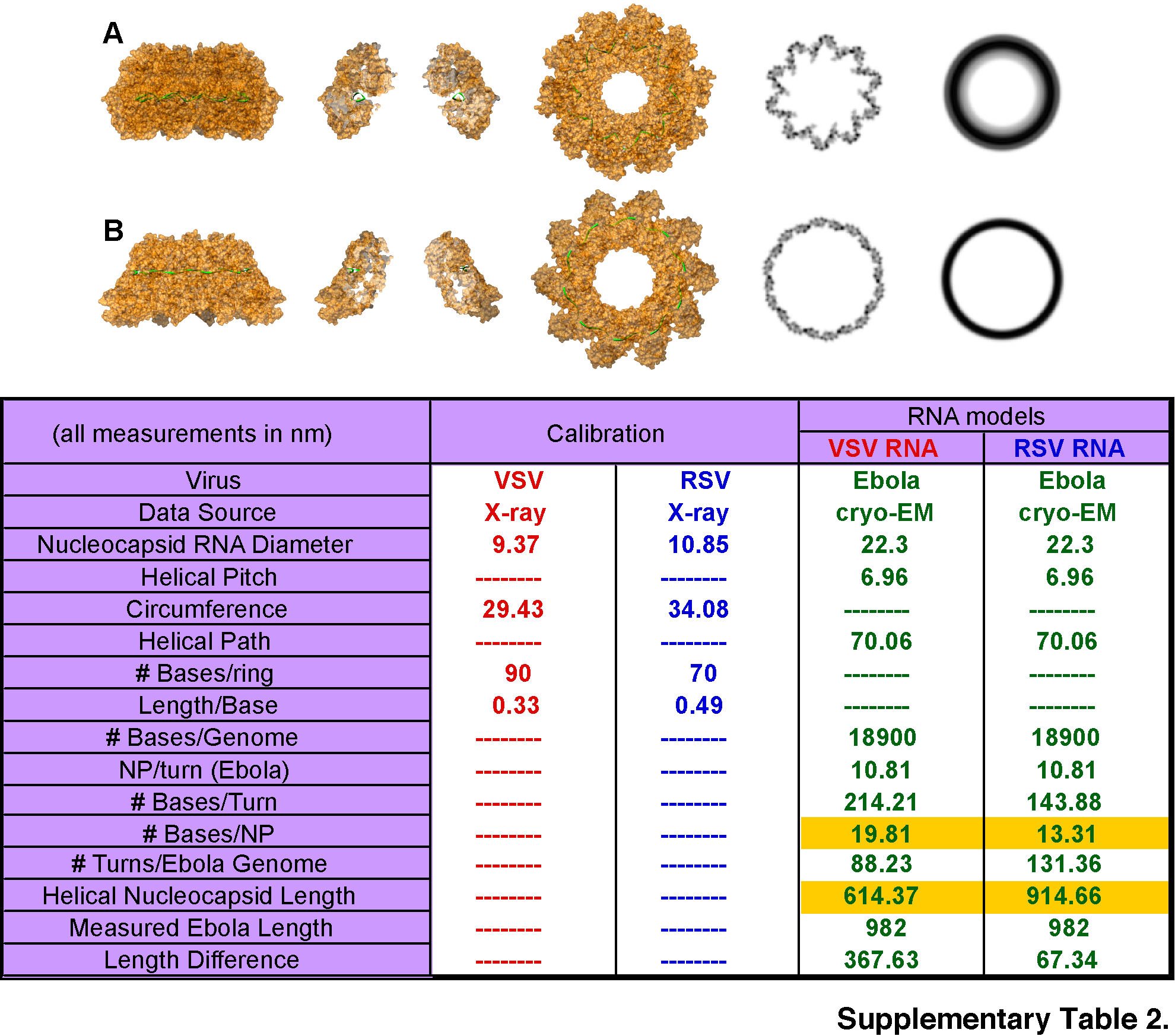

Supplement: Table S2 — Modeling of RNA in the Ebola nucleocapsid. Two previously determined atomic resolution structures of negative stranded RNA viruses (VSV (2GIC.pdb) [58], and RSV (2WJ8.pdb) [32]) were used to estimate the EBOV nucleocapsid length and number of nucleotides per nucleoprotein. Images of VSV (A) and RSV (B) are shown as a molecular surface with the protein in orange and the RNA as a green ribbon. From left to right, they show a surface view from the side, a side-on cross section, an end-on view, the RNA density alone in projection, and a rotational average of the projection. The VSV-based estimate, with a saw-tooth pattern of RNA in the helix, gave a nucleocapsid which 614.37 nm long, too short for the measured length of the G1 EBOV (982 nm). The RSV-based model, with a relatively straight/circular pattern of RNA in the helix predicted a nucleocapsid 914.55 nm long, which closely fits the measured length of G1 virions, after allowing ∼34 nm space at each end to accommodate the curve of the envelope containing GP spikes and matrix proteins. The RSV-like model gives 13 nucleotides per nucleocapsid protein which is similar to previous biochemical estimates of 12–15 for Marburg virus [33], suggesting that the RNA in the EBOV nucleocapsid is arranged in a smooth helical pattern at a diameter of ∼22 nm. (TIF) [file pone.0029608.s007.tif]
